# Spermidine overrides INSR (insulin receptor)-IGF1R (insulin-like growth factor 1 receptor)-mediated inhibition of autophagy in the aging heart

**DOI:** 10.1080/15548627.2022.2095835

**Published:** 2022-07-10

**Authors:** Mahmoud Abdellatif, Frank Madeo, Guido Kroemer, Simon Sedej

**Affiliations:** a Department of Cardiology, Medical University of Graz, Graz, Austria; b Centre de Recherche des Cordeliers, Equipe labellisée par la Ligue contre le cancer, Université de Paris, Sorbonne Université, Inserm U1138, Institut Universitaire de France, Paris, France; c Metabolomics and Cell Biology Platforms, Institut Gustave Roussy, Villejuif, France; d BioTechMed Graz, Graz, Austria; e Institute of Molecular Biosciences, University of Graz, Graz, Austria; f Field of Excellence BioHealth, University of Graz, Graz, Austria; g Institut du Cancer Paris CARPEM, Department of Biology, Hôpital Européen Georges Pompidou, AP-HP, Paris, France; h Institute of Physiology, Faculty of Medicine, University of Maribor, Maribor, Slovenia

**Keywords:** Heart failure, human, IGF1R, insulin signaling, longevity, mitochondrial dysfunction, mouse, PI3K

## Abstract

Although attenuated IGF1R (insulin-like growth factor 1 receptor) signaling has long been viewed to promote longevity in model organisms, adverse effects on the heart have been the subject of major concern. We observed that IGF1R is overexpressed in cardiac tissues from patients with end-stage non-ischemic heart failure, coupled to the activation of the IGF1R downstream effector AKT/protein kinase B and inhibition of ULK1 (unc-51 like autophagy activating kinase 1). Transgenic overexpression of human IGF1R in cardiomyocytes from mice initially induces physiological cardiac hypertrophy and superior function, but later in life confers a negative impact on cardiac health, causing macroautophagy/autophagy inhibition as well as impaired oxidative phosphorylation, thus reducing life expectancy. Treatment with the autophagy inducer and caloric restriction mimetic spermidine ameliorates most of these IGF1R-induced cardiotoxic effects in vivo. Moreover, inhibition of IGF1R signaling by means of a dominant-negative phosphoinositide 3-kinase (PI3K) mutant induces cardioprotective autophagy, restores myocardial bioenergetics and improves late-life survival. Hence, our results demonstrate that IGF1R exerts a dual biphasic impact on cardiac health, and that autophagy mediates the late-life geroprotective effects of IGF1R inhibition in the heart.

Reducing the activity of the IGF1 (insulin-like growth factor 1) pathway extends the lifespan of various model organisms. However, IGF1 is also involved in cellular growth and metabolism of almost all organs. Thus, it remains controversial as to whether IGF1 signaling promotes or protects from age-related, non-proliferative pathologies. To this end, conflicting evidence has emerged from preclinical and human studies regarding the relationship between IGF1 signaling and cardiac health, indicating a potential uncoupling between cardiac health and lifespan in response to IGF1 signaling inhibition. In support of this notion, mice with cardiomyocyte-specific overexpression of human IGF1R exhibit increased cardiac muscle mass and superior function, a phenotype often described to be reminiscent of exercise-induced myocardial hypertrophy in athletes. However, this view has been recently challenged, because aged mice treated with IGF1R monoclonal antibodies and mice lacking IGF1R in cardiac myocytes do not show a deleterious cardiac phenotype later in life. To resolve these conflicting observations and to better define the role of the IGF1R signaling pathway in cardiac aging, we performed a lifelong study, in which we resolved time-dependent effects of the IGF1R pathway in mice with enhanced versus reduced IGF1R signaling specifically in cardiac myocytes [1].

In our study, we demonstrated that young male mice with cardiomyocyte-specific overexpression of human IGF1R (IGF1R*^tg^*) exhibit enhanced cardiac function and exercise capacity. However, during the course of aging, these benefits gradually fade, and late-in-life IGF1R*^tg^* mice develop clear signs of heart failure, compromising ejection fraction, cardiac output and cardiopulmonary capacity, accompanied by left atrial dilation and pulmonary congestion, resulting in reduced life expectancy. Mechanistically, aged IGF1R*^tg^* hearts exhibit increased collagen accumulation, impaired mitochondrial function and reduced resistance to oxidative stress. Importantly, all these typical features of heart failure are preceded by reduced autophagic flux in the heart, suggesting that autophagy blockade might be responsible for the deleterious cardiac phenotype in aged IGF1R*^tg^* mice. In fact, late-in-life treatment with the autophagy inducer and caloric restriction mimetic spermidine preserves systolic function, prevents atrial dilation and improves markers of oxidative phosphorylation in aged IGF1R*^tg^* mice. Hence, reduced autophagy might causally mediate the late-life detrimental impact of chronically elevated IGF1R signaling in the heart.

To further corroborate the biphasic role of IGF1R signaling in the heart, we examined mice with reduced IGF1R signaling activity due to a mutation in *Pik3ca/p110α*, the gene encoding the phosphoinositide 3-kinase (PI3K) catalytic subunit, which is a key downstream effector of IGF1R. Unlike young IGF1R*^tg^* mice, age-matched mice with a dominant-negative PI3K (dnPI3K) isoform in cardiac myocytes exhibit delayed cardiac growth, as indicated by lower cardiac mass, ejection fraction and exercise capacity. In stark contrast, old dnPI3K mice have clearly attenuated cardiac aging, characterized by preserved systolic function and enhanced myocardial energy reserves. Aged dnPI3K mice also show reduced age-related hypertrophic remodeling and extended longevity. The cardiac benefits in dnPI3K mice correlate with activated autophagic flux, while treatment with the lysosomotropic agent hydroxychloroquine, which blocks the fusion of autophagosome with lysosomes, aggravates cardiac function, indicating that functional autophagy is indispensable for the geroprotective effects of reduced IGF1R-PI3K signaling in the heart.

To determine the clinical relevance of these experimental observations, we examined IGF1R expression and signaling activity in human ventricular samples of explanted hearts with end-stage non-ischemic heart failure, which were compared to age-matched donor hearts with no history of cardiac disease or with compensated hypertrophy. At variance with non-failing hearts, explanted failing hearts exhibit increased IGF1R expression and signaling activity, as indicated by increased phosphorylation of the serine/threonine-protein kinase AKT. Intriguingly, among the downstream targets of IGF1R, we detect increased MTOR-dependent ULK1 phosphorylation, indicative of autophagy inhibition, but no alterations in RPS6KB1/p70 S6K1 (ribosomal protein S6 kinase B1) phosphorylation. Of note, compensated cardiac hypertrophy is not associated with clear shifts in IGF1R expression or signaling activity.

In summary, despite the pro-longevity impact of low IGF1R signaling in model organisms, the consequences of reduced IGF1R signaling on cardiac aging remained hitherto controversial. Such uncoupling of healthspan and lifespan has limited the enthusiasm for inhibiting IGF1R signaling as a valid strategy to delay aging in humans. Our study brings into question this assertion, as we discovered that late-life and cell-specific targeting of IGF1R signaling might be therapeutically harnessed for anti-aging benefits. Through a series of clinical and experimental studies, we found that the relationship between IGF1R signaling and cardiac function is not linear as previously thought, but rather biphasic and age-dependent. This finding challenges the classical view of the role of IGF1 signaling in cardiac aging and suggests a novel paradigm for therapeutic targeting of IGF1R signaling, thereby considering age as a major outcome-determining factor. While IGF1R inhibition is detrimental in early life for the growth and function of the heart, targeting IGF1R signaling during late-life phases might exert cardioprotective effects, at least in part through autophagy induction (Figure 1). Hence, autophagy inducers, such as spermidine, as well as pharmacological inhibitors of IGF1R or PI3K, which are already clinically used against cancer, should be evaluated for their potential efficacy to improve cardiac health and extend lifespan in elderly patients at risk of heart failure.

**Figure 1.**
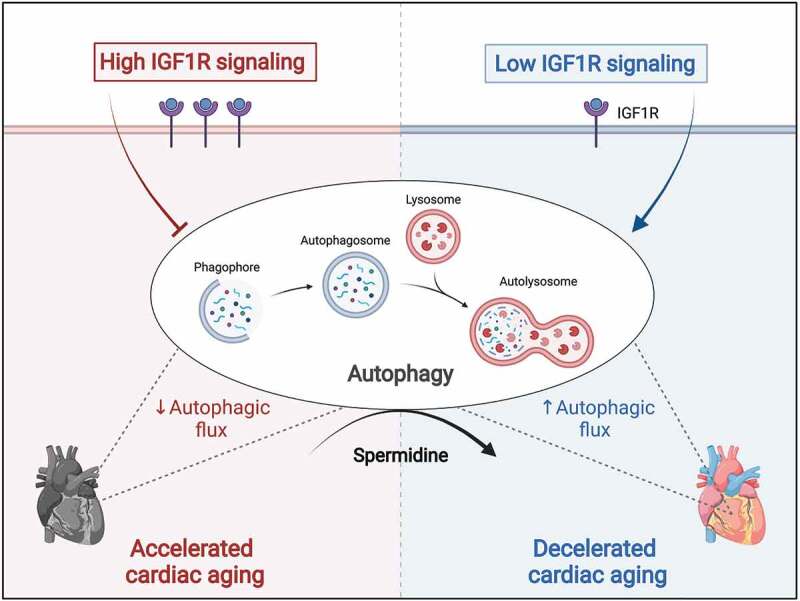
Autophagy determines the effect of IGF1 (insulin-like growth factor 1) signaling on the aging heart. In mice, cardiomyocyte-specific overexpression of human IGF1R (IGF1 receptor) accelerates cardiac aging, leading to premature heart failure, despite initially inducing physiological cardiac hypertrophy and superior function. Treatment with the autophagy inducer spermidine protects aged IGF1R*^tg^* mice from heart failure, suggesting that reduced autophagic flux underlies the late-life detrimental impact of increased cardiac IGF1R signaling. By contrast, low IGF1R signaling in mice harboring a dominant-negative mutation in the PIK3CA/p110α isoform of phosphoinositide 3-kinase (PI3K) – which is a key downstream effector of IGF1R – decelerates cardiac aging and extends longevity in an autophagy-dependent manner, as indicated by limited cardioprotection upon treatment with the autophagy inhibitor hydroxychloroquine. This figure was created with BioRender.com.

## Supplementary Material

Supplemental Material
