# Supplementary material for: Spermidine overrides INSR (insulin receptor)-IGF1R (insulin-like growth factor 1 receptor)-mediated inhibition of autophagy in the aging heart
Source: Autophagy. 2022 Jul 10;18(10):2500–2. doi: 10.1080/15548627.2022.2095835 (PMC9542397; doi:10.1080/15548627.2022.2095835)
Supplement: Supplemental Material [file KAUP_A_2095835_SM7325.zip › IGF autophagy final.pdf]

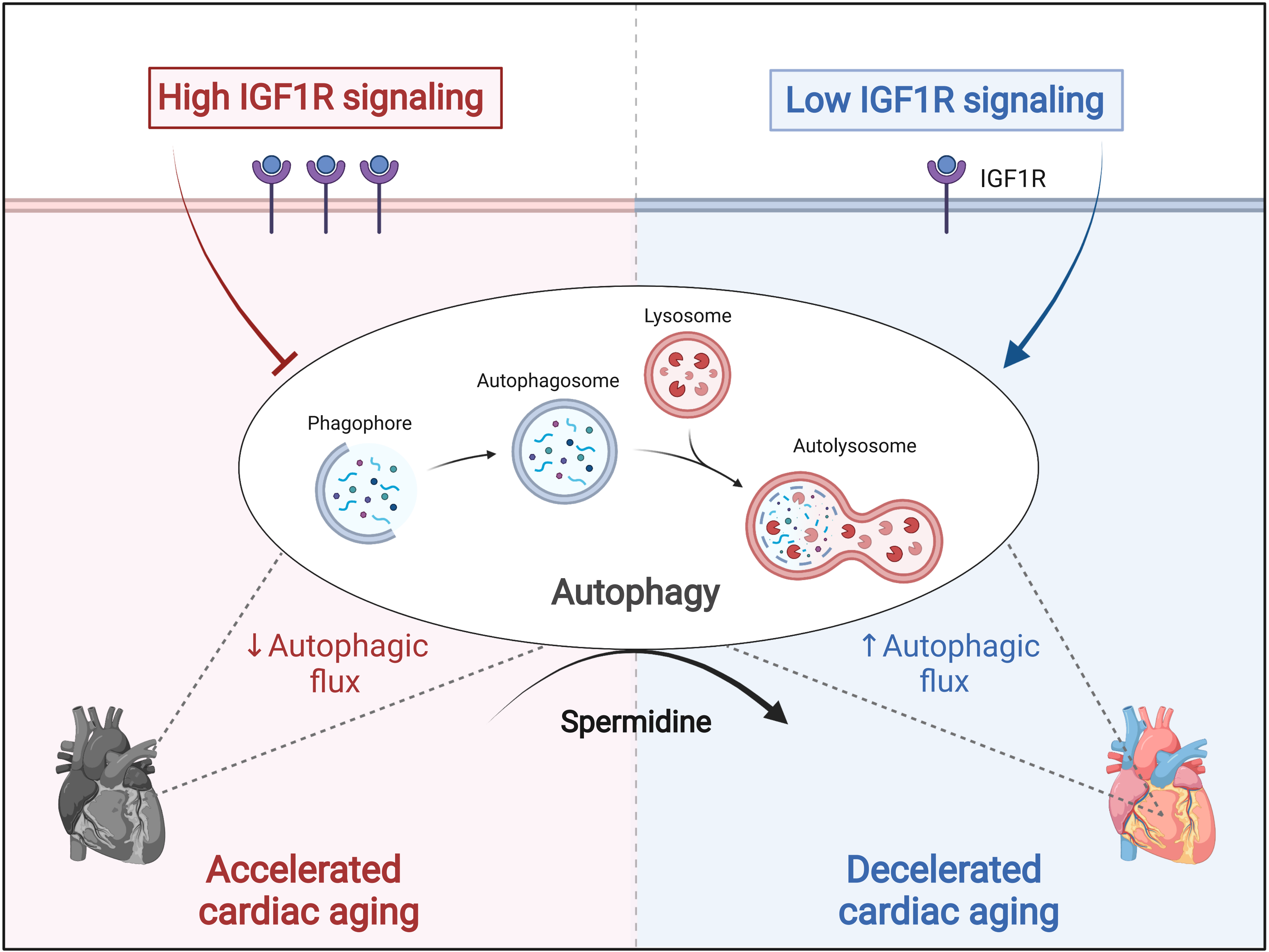
High IGF1R signaling
Low IGF1R signaling
IGF1R
Lysosome
Autophagosome
Phagophore
Autolysosome
Autophagy
↓Autophagic flux
↑Autophagic flux
Spermidine
Accelerated cardiac aging
Decelerated cardiac aging
